# Supplementary material for: Empirical methods for controlling false positives and estimating confidence in ChIP-Seq peaks
Source: BMC Bioinformatics. 2008 Dec 5;9:523. doi: 10.1186/1471-2105-9-523 (PMC2628906; doi:10.1186/1471-2105-9-523)
Supplement: Additional File 1 — A variety of html documents from the USeq web site detailing the available applications, their best usage, output file type descriptions, command line menus, etc. [file 1471-2105-9-523-S1.zip › USeqUserGuides/JavaDoc.zip!/JavaDoc/util/bio/annotation/class-use/ExonIntron.html]

Uses of Class util.bio.annotation.ExonIntron


|  |  |  |
| --- | --- | --- |
| **Overview** \| **Package** \| **Class** \| **Use** \| **Tree** \| **Deprecated** \| **Index** \| **Help** | |  |
| PREV   NEXT | **FRAMES**    **NO FRAMES**     **All Classes** |  |


---


## **Uses of Class util.bio.annotation.ExonIntron**

| Packages that use ExonIntron | |
| --- | --- |
| **gata.main** | Main app, utility, and params for GATAPlotter. |
| **trans.main** | Principle classes and scripts used in TiMAT2 analysis. |
| **util.bio.annotation** | For representing gene annotation. |
| **util.bio.parsers** | Parsers and extractors, fasta, quality, text, gff.... |

Uses of ExonIntron in gata.main

| Methods in gata.main with parameters of type ExonIntron | |
| --- | --- |
| `static java.util.ArrayList` | `GATAUtil.extractStartEnds(ExonIntron[] exons)`             Deep copies start stops from ExonIntron objects to an ArrayList |
| `static int[][]` | `GATAUtil.extractStartEndsInts(ExonIntron[] exons)` |

Uses of ExonIntron in trans.main

| Methods in trans.main with parameters of type ExonIntron | |
| --- | --- |
| `int[][]` | `ScanGenes.convertToIndexPositions(ExonIntron[] exons)`             Converts an array of ExonIntron to an int[exon number][start index in bp positions array, stop index] |

Uses of ExonIntron in util.bio.annotation

| Methods in util.bio.annotation that return ExonIntron | |
| --- | --- |
| `ExonIntron[]` | `TransGroup.getExons()` |

| Methods in util.bio.annotation with parameters of type ExonIntron | |
| --- | --- |
| `static java.util.ArrayList` | `GeneGroup.extractStartEnds(ExonIntron[] exons)` |
| `static int[][]` | `GeneGroup.extractStartEndsInts(ExonIntron[] exons)` |

Constructors in util.bio.annotation with parameters of type ExonIntron

`TransGroup(Transcript transcript,
Translation translation,
ExonIntron[] exons)`

Uses of ExonIntron in util.bio.parsers

| Methods in util.bio.parsers that return ExonIntron | |
| --- | --- |
| `ExonIntron[]` | `UCSCGeneLine.getExons()` |

| Methods in util.bio.parsers with parameters of type ExonIntron | |
| --- | --- |
| `void` | `UCSCGeneLine.setExons(ExonIntron[] exons)` |

---


|  |  |  |
| --- | --- | --- |
| **Overview** \| **Package** \| **Class** \| **Use** \| **Tree** \| **Deprecated** \| **Index** \| **Help** | |  |
| PREV   NEXT | **FRAMES**    **NO FRAMES**     **All Classes** |  |


---
